# Antipredatory strategies of terrestrial isopods

**DOI:** 10.3897/zookeys.1101.76266

**Published:** 2022-05-18

**Authors:** Ivan Hadrián Tuf, Barbora Ďurajková

**Affiliations:** 1 Department of Ecology and Environmental Sciences, Faculty of Science, Palacký University, Olomouc, Czech Republic Palacký University Olomouc Czech Republic

**Keywords:** Aggregation, aposematism, behavioural traits, death feigning, defence, mimicry, Oniscidea

## Abstract

Terrestrial isopods (Oniscidea) represent a widespread group of land Crustacea that have been able to successfully adapt to the terrestrial environment and occupy newly formed ecological niches. During the colonisation of land, they faced numerous challenges, including finding an effective way to avoid their new terrestrial predators. In response to predation pressure, they have developed various behavioural and morphological adaptations. These include tonic immobility, conglobation, clinging to the ground, releasing strongly acidic secretions, jumping, and efficient running away. Furthermore, terrestrial isopods can aggregate with other individuals, use stridulation, or change their typical activity time. Some of them also developed spiny tergites and aposematic colouration or posture. The majority of these strategies have not yet been studied.

## Introduction

Oniscidea, commonly called terrestrial isopods, woodlice or pill bugs, represent one of the eleven suborders belonging to the order Isopoda (Pericarida, Crustacea) that first appeared on land during the lower Carboniferous (Schram 1982; Fu et al. 2010). According to Broly et al. (2013), the oldest fossils of Oniscidea come from the Early Cretaceous, but some indications suggest that they could have already appeared in the pre-Pangaean period, most likely in the Carboniferous interval of the Late Palaeozoic. They were able to colonise various terrestrial habitats ranging from sea level to high mountains (Hornung 2011), and are represented by ~ 4,000 species distributed in more than 500 genera and 38 families (Javidkar et al. 2015; Sfenthourakis and Taiti 2015; Dimitriou et al. 2019; WoRMS 2021). Thus, Oniscidea represents the most flourishing crustacean group that has ever colonised the land. As mentioned in many previous studies, oniscid isopods must evolve various adaptations for their terrestrial life. They had to solve ecological and physiological problems, such as respiration, feeding, locomotion, reproduction, and at the same time protect themselves against desiccation in their new terrestrial habitats (Hornung 2011).

As terrestrial isopods colonised land some 300 MA, they faced several predators, such as centipedes, spiders, amphibians, and reptiles. Predator pressures caused Oniscidea to develop various morphological and behavioural adaptations. The lifespan of terrestrial isopods ranges from 1 to ~ 10 years. The highest mortality rate is within the first month of their life outside the brood pouch; this is most frequently due to climate factors, such as high temperatures, drought, and floods, as well as due to cannibalism of different ontogenetic stages (Brereton 1957). As previously mentioned, terrestrial isopods are the prey of a wide variety of predators. Additionally, for some of them (e.g., small passerines such as flycatchers or wrens), terrestrial isopods are a main source of calcium (Bureš 1986; Krištín 1992). Also, there are specialists, such as *Scorpiomauruspalmatus* (Ehrenberg, 1828), whose major prey consists of the desert isopod *Hemilepistusreaumurii* (Milne Edwards, 1840) at some localities (Warburg 1993). Other well-known specialists are spiders of the genus *Dysdera* (Řezáč and Pekár 2007), with numerous species using at least three different hunting strategies by means of their specifically adapted chelicerae.

Predators keep up with antipredator adaptations of prey and improve their own hunting techniques accordingly. This never-ending struggle causes the creation of numerous adaptations of both predators and prey, i.e., terrestrial isopods. Although the various types of antipredation strategies among animals are well known, a comprehensive overview on the subject concerning terrestrial isopods is lacking. Therefore, this text provides a systematic review of currently known information regarding the antipredation strategies of terrestrial isopods. The known methods of terrestrial isopod defence against predators, both behavioural as well as morphological, are summarised below, including tentatively proposed strategies.

## Not to be there

The simplest strategy to defend oneself against a predator is to keep one’s distance from it, and to avoid an encounter with it. A useful strategy is to avoid staying in places where other individuals were killed. Supporting a necromone hypothesis, Yao et al. (2009) experimentally confirmed that terrestrial isopods are deterred by chemical substances (mainly linoleic acid) released by smashed terrestrial isopods (mainly their conspecifics).

In general, movement in a linear path represents the most efficient adaptive escape strategy when precise information about environmental risks for an animal is lacking (Jander 1975). In an open space, terrestrial isopods like *Armadillidiumvulgare* (Latreille, 1804) move forward in a straight line (Iwata and Watanabe 1957). When *A.vulgare* and *Porcelliolaevis* (Latreille, 1804) were kept in a box with the ants *Tetramoriumcaespitum* (Linnaeus, 1758), both species escaped the shelters and were more active outside of them as a result of ant harassment. The same effect was caused by an indirect cue from the predatory ants: both species of isopod kept themselves further away from the source of the ant odour (Castillo and Kight 2005).

As terrestrial isopods usually live on the soil surface, it is difficult for them to walk straight and maintain their direction while walking around numerous obstacles (e.g., stones, pebbles, and vegetation stems). The most effective way for them to keep a straight-forward direction is through systematic turn alternations (Hughes 1967). This is the way to reach the greatest distance from a starting point with the highest level of probability (Hughes 1989; Moriyama and Migita 2004). This behavioural pattern is shared by a diverse range of animals, including humans (Lepley and Rice 1952; Grosslight and Harrison 1961; Pate and Bell 1971).

A sophisticated method for testing the stress of terrestrial isopods is by keeping track of their movements through a T-maze (Ono and Takagi 2006). Terrestrial isopods use turn alternation as their strategy to escape from unpleasant places, and the intensity of the turn alternation is used to evaluate their level of stress (Houghtaling and Kight 2006). *Porcellioscaber* Latreille, 1804 more intensively alternated turns when they met a spider (e.g., the dangerous *Dysderacrocata* CL Koch, 1838) than when they met a harmless fly or cotton flock (Carbines et al. 1992). Chronic stress induced by indirect predatory cues (e.g., ant odour) can lead to increased turn alternation in *P.laevis* and *A.vulgare* (Hegarty and Kight 2014), behaviour that leads to the desertion of a dangerous place. Also, these isopods can use small chronical substrate vibrations for the detection of a predator (Zimmerman and Kight 2016). Habituation to the disturbance can significantly reduce turn alternation (Houghtaling and Kight 2006). The similarity in turn alternation of distantly related species of terrestrial isopods suggests evolutionary conservation of these antipredator mechanisms (Hegarty and Kight 2014). It is also known that this escape behaviour is a result of the isopod’s own decision-making (Moriyama 1999; Moriyama and Migita 2004) and that *A.vulgare* can correct its turns to increase its level of alternation (Moriyama et al. 2016).

Terrestrial isopods not only engage in spatial predator avoidance, but they also engage in temporal predator avoidance. Terrestrial isopods can avoid encounters with a predator by changing the time range in which they are active. For example, while most terrestrial isopods are nocturnal, the burrowing isopods *H.reaumurii*, from the arid regions of North Africa, the Middle East, and Central Asia, are active in the colder season during full daylight (Nasri-Ammar and Morgan 2005). During the warmest months when the temperature can increase to 45 °C, *H.reaumurii* becomes active before sunrise, thereby exposing themselves to *Scorpiomaurus*, their main nocturnal predator. Therefore *H.reaumurii* can switch its typical terrestrial isopod activity from night to day for the sake of reducing the rate of its predation by scorpions. Although no study confirming this claim has been published yet, similar behaviour has been observed in other animals. For example, European rabbits have switched from nocturnal to diurnal activity after the appearance of European polecats, a typical night-time predator, in an area where rabbits were already present (Bakker et al. 2005).

## Not to be seen

The tendency “not to be seen” can be an escape mechanism from predators. Some terrestrial isopod species, the so-called “runners” group according to the ecomorphological classification of Schmalfuss (1984), have well-developed eyes and a relatively narrow body with long pereiopods; these traits make them suitable for a quick escape (Schmalfuss 1984). For example, *Philosciamuscorum* (Scopoli, 1763) is well adapted for a fast and surprising retreat thanks to its slim body and long legs (Sutton 1972). An astonishing antipredatory strategy related to “not to be seen” is diving (Leistikow 2001). An example is *Ischiosciahirsuta* Leistikow, 2001, which can hide under the water surface of small streams if disturbed.

A jumping strategy is more unpredictable than a running strategy for a hunter. For several species of Philoscia (Ischioscia), jumping, akin to springtails (Collembola), was reported (Williams 1941). Van Name (1925, 1926) mentioned leaping or jumping for *Ischioscianitida* (Miers, 1877, but considered as nomen dubium) and *Ischiosciavariegata* (Dollfus, 1893). Leistikow (2001) compared the jumping distance of *I.variegata* and *Ischiosciapariae* Leistikow, 2001 with each other. He described how the first species was recorded jumping up to 20 cm, while the second was recorded only jumping ~ 5 cm. Such distances should be far enough to avoid hunters (e.g., a spider). Leistikow (2001) also mentioned that a 20 cm-long jump was sufficient to escape human collectors. Jumping terrestrial isopods were reported also from Borneo, a species of *Burmoniscus*, and were subsequently assigned to the ecomorphological type “jumper” (Cranbrook and Edwards 1994; Hassall et al. 2006); this, despite the fact that jumpers and runners differ only in behaviour.

Visually oriented larger predators, such as amphibians, lizards, or birds, are attracted by the movement of prey. A very simple strategy related to “to not be seen”, not only used by terrestrial isopods, is to stay inactive when disturbed. When *A.vulgare* and *P.laevis* detect a predatory spider, they reduce their activity as a response (Zimmerman and Kight 2016). This behavioural strategy is typical for species of the ecomorphological group “clingers” (Schmalfuss 1984), which have short strong legs, and can cling firmly to the substratum. Their dorsal part is protected by a strong exoskeleton that has broad tergites that expand their body shape, making it impossible to catch them or to turn them when clinging onto the substrate. Another strategy which is, for instance, characteristic for species living on tree bark is to fall down. When a bird tries to detach a terrestrial isopod from a bark, there is a high probability that the isopod will fall off the tree and become lost in the leaf litter around the base of the tree trunk (cf. Butler 1889). The subsequent immobility causes invisibility by the cryptic colouration of the terrestrial isopods, stained by different shades of grey, brown, and beige (Achouri and Charfi-Cheikhrouha 2009).

The tendency of terrestrial isopods to not instigate predators by their movement can also be related to “tonic immobility”. This is the state of reversible physical immobility and muscle hypertonicity during which the animals do not respond to external stimuli (Gallup 1974). Immobility is an often-used form of passive anti-predator behaviour adopted by a wide scale of animals, including terrestrial isopods (Quadros et al. 2012; Tuf et al. 2015). Tonic immobility is not a simple synonym of death feigning, i.e., thanatosis. Thanatosis is not necessarily tonic, such as in invertebrates or opossums (Francq 1969); it can also be in a relaxed state, such in some birds, mammals, or snakes (Holmes 1916). Moreover, the typical posture of a tonically immobile individual is usually unlike the posture of a genuinely dead individual, as mentioned by Darwin (cf. Holmes 1916). Typical tonic immobility posture of the clinger ecomorphological group of terrestrial isopods was described by Quadros et al. (2012) as follows: “The contraction of the body to form a comma-like shape and the contraction and folding of the legs towards the ventral side while holding the antennae folded or extended backward and pressed against the dorsal contour of the first pereonites”. Terrestrial isopods of the ecomorphological group “rollers” (Schmalfuss 1984) adopt specific ball-like postures, this behaviour is called conglobation (or volvation, cf. Verhoeff 1930).

While adopting a posture increases the protection of an animal against being swallowed by a predator (Tuf et al. 2015), feigning death reduces the probability of being seen by predators. Thus, tonic immobility is a defence strategy against visually oriented predators. There are also some indications that the duration of thanatosis depends on the daily light regime. This was recorded in the freshwater crab *Trichodactyluspanoplus* (von Martens, 1869) (Zimmermann et al. 2009) and the coleopteran *Cylasformicarius* (Fabricius, 1798) (Miyatake 2001). Another factor that can influence responsiveness to tonic immobility is temperature (Miyatake et al. 2008). Additionally, the type of stimulus can influence responsiveness, as was proved by Quadros et al. (2012) in their study of three terrestrial isopods *Balloniscusglaber* Araujo & Zardo, 1995, *Balloniscussellowii* (Brandt, 1833), and *Porcelliodilatatus* Brandt, 1833. The duration of tonic immobility varies intraspecifically, and is related to the survival probability of prey. The antipredator behaviour of terrestrial isopods can be age-dependent, and may change during their life course. For example, *B.sellowii* uses tonic immobility more often when young and small when compared with older and larger individuals that employ more active escape strategies, such as running (Quadros et al. 2012). Body size can also play a crucial role in the effectiveness of tonic immobility because smaller animals are more likely to be disregarded by a predator. The discrepancy between escape and tonic immobility, both effective strategies, can lead to distinguishable personalities of terrestrial isopods, as shown for *P.scaber* (Tuf et al. 2015). Thus, terrestrial isopods can increase their survivorship using tonic immobility in one of two ways: they can either increase their resemblance with the surrounding environment and be less visible (Bergey and Weis 2006), or they can protect their vulnerable ventral surface (Quadros et al. 2012).

## Not to be bitten

The soft vulnerable ventral surface of any terrestrial isopod is best protected during conglobation, which allows them to survive in conditions that may be lethal to other species (White and Zar 1968). This behaviour can be found among mammals, such as pangolins, hedgehogs, echidnas (Sigwart et al. 2019), tenrecs, and armadillos. It is also typical for arthropods such as pill millipedes, giant pill millipedes, soil mites, cuckoo wasps (Tuf et al. 2015), multi-shelled chitons (Eernisse et al. 2007), beetles (Ballerio and Grebennikov 2016), cockroaches (Perry and Nalepa 2003), trilobites, and some larvae of other groups (Haug and Haug 2014). This tonically immobile posture is typical among members of the families Armadillidae, Armadillidiidae, Cylisticidae, Tylidae, Helleriidae, Buddelundiellidae, Scleropactidae, or Eubelidae.

The ability to conglobate depends on several body characteristics. The bend of the tergites and the ventral muscles are the most important features that enable conglobation. Aside from an animal’s arched shape, there are a wide range of additional body part adaptations common for species capable of conglobation. These include the shape of the head, the shape and length of the antennae, the shape of the epimers of the pereonites, and the shape of the pleon, telson, and uropods. Species with the ability to roll up have often developed head grooves in which the antennae can fit (Sutton 1972). Also, conglobation leads to some adaptations of the female´s marsupium (a brood pouch in peracarid crustaceans). In ovigerous females, the oostegites allow them to bend enough to conglobate (Csonka et al. 2015). As consequence of conglobation, the female’s internal organs are compressed and displaced, with the eggs are condensed into the anterior part of the marsupium. As a result, females may stop feeding themselves in advanced stages of gravidity (Appel et al. 2011). Although the marsupium of conglobating species does not protrude as in non-conglobating species, in the last days of the incubation of mancas it can prevent perfect conglobation. The length of the breeding period can be shortened due to the presence of predators (Castillo and Kight 2005), which is indirect support that conglobation is an antipredatory strategy. Additionally, due to the smooth surface of the conglobated isopod, it is more difficult for predators to find a suitable place for attack (Řezáč et al. 2008).

Conglobation is usually triggered by external stimuli, such as strong vibrations or pressure (Horváth et al. 2019). Cividini and Montesanto (2018a) documented that *Armadilloofficinalis* Duméril, 1816 responded to substrate vibrations by conglobation. Even against larger, visually oriented predators, conglobation can be a useful adaptation since a ball-like body can roll away and disappear into leaves or debris. This is more important for non-perfect conglobation (typical of the genus *Cylisticus*), because the uropods and antennae are not well protected. Anecdotally, it is cruelly ironic that the typical ball-like shape of a defending *A.vulgare* was the only reason that humans ate them (giving the name of “pill-bug” to all conglobating terrestrial isopods) – its antipredatory strategy was, in this case, a reason for higher predatory pressure (Duméril 1816).

Although intraspecific variability in the use of tonic immobility in the “clinger” species *P.scaber* is high (Tuf et al. 2015), conglobation used by rollers is more constant (Matsuno and Moriyama 2012; Cazzolla Gatti et al. 2019). In addition to its function for protection against predators, terrestrial isopods can use conglobation to limit water loss (Smigel and Gibbs 2008).

## Not to be alone

If a prey species is distributed homogenously, it is easier for a predator to encounter prey frequently and eat ad libitum; therefore, a very simple antipredatory strategy is for prey to be grouped together. Aggregation into groups is considered an evolutionarily successful response to predator pressure, ambient temperature, and water deficits (Broly et al. 2013). The advantages of living in aggregations were described by Allee (1926), and positive density dependency, or the positive correlation between population density and individual fitness, is called the “Allee effect” (Krause and Ruxton 2002). As Krause and Ruxton (2002) pointed out, aggregative defensive behaviour is a common response to the risk of predation, and is widespread among a diverse range of animal groups.

One of the basic characteristics of this type of defensive behaviour is the predator confusion. People who have tried to collect aggregated isopods can confirm that individually handling them can give the majority of isopods enough time to disappear. Even a skilled predator is not capable of eating all individuals in a group. The size of the group has an inverse correlation with the probability that a particular individual in the group will be attacked; i.e., the larger the group, the less likely it is for an individual to be attacked. Actually, a higher visibility of large groups of prey, i.e., a higher attack rate per group, is less important for each individual than is the much lower probability of being eaten while “hiding” in large groups (Krause and Ruxton 2002).

In aggregations, information about an approaching attack can be transmitted from individuals who did observe the danger to those who have not yet noticed it. Such behaviour was documented in *A.officinalis*, which can produce substrate vibrations to warn neighbouring individuals (Cividini et al. 2020). In addition, aggregation can intensify the effect of individual defence mechanisms, such as repulsive secretions or necromones (chemical compounds released by dead terrestrial isopods, cf. Yao et al. 2009), and thus functions as a shared defence behaviour (Broly et al. 2013). The study of Cividini and Montesanto (2018a) proved that the isopod’s response to micro-vibrations leads to a greater number of aggregates, considering that micro-vibrations can warn for an impending danger. Aggregation behaviour in terrestrial isopods is thoroughly studied (Broly et al. 2012, 2013, 2014, 2016; Broly and Deneubourg 2015; Pogson 2016); however, a comprehensive study of the impact of aggregation on predation risk in terrestrial isopods is yet to be done.

Only one terrestrial isopod, *A.officinalis*, is known to produce sounds that are audible to humans. This sound is produced by stridulation through a ledge of scales situated on the propodus of the fourth and fifth pereopod (Caruso and Costa 1976). This feature is present in both sexes (Taiti et al. 1998) from the early stages of development onwards (Montesanto 2018) and occurs in all species of *Armadillo* (Schmalfuss 1996). Although terrestrial isopods do not have a sense of hearing, they can register substrate-borne vibrations caused by their stridulation (Cividini and Montesanto 2018a, b). *Armadilloofficinalis* responds to substrate vibrations by conglobation or by deviating from the source of vibration, although juveniles usually conglobate (Cividini and Montesanto 2018c). This response may be caused by the perception that these vibrations are a sign of danger (Zimmerman and Kight 2016). Escape behaviour in response to vibrations was also noted in *A.vulgare* by Moriyama (2004), and *P.laevis* systematically alternates its turns in a T-maze in response to vibrations when not habituated to the vibrations beforehand (Houghtaling and Kight 2006). Although no sensory receptor in terrestrial isopods has been reported yet, the high sensitivity of *A.vulgare*, *P.laevis* as well as *A.officinalis* to vibrations suggests its presence.

Additionally, substrate-borne vibrations induced by stridulations can be a strategy of intraspecific communication. The pill bug *A.officinalis* can probably warn other individuals of imminent danger and adverse conditions, and thus ensures a higher survival rate of neighbouring individuals (Cividini et al. 2020). Indeed, its response to micro-vibrations is an intensification of aggregation behaviour (Cividini and Montesanto 2018a). Perhaps *A.officinalis* can also use stridulation during mating to convince females to uncoil and mate, as the morphologically similar deaf giant pill millipedes (Sphaerotheriida) do (Wesener et al. 2011); however, this topic has not been studied yet.

Stridulation could also work as an antipredatory strategy. In giant pill millipedes (Sphaerotheriida) this function of stridulation was reported more than a hundred years ago (Gravely 1915) as protecting *Arthrosphaeraaurocincta* Pocock, 1899 against reduviid bugs of the genus *Physorhynchus*, but without a description of the mechanism. As in the case of *A.officinalis* (Cividini et al. 2020), these millipedes are only able to stridulate during the conglobation (the antipredator behaviour mentioned above). Thus, stridulation, as a secondary form of defence, can be used to discourage predators more effectively. These millipedes stridulate in response to handling (Gravely 1915) in a similar way to *A.officinalis*.

Defensive sounds could similarly be aposematic; that is, they could be the acoustic counterparts of visual aposematic signals, differing only in the way that they can fulfil their function both in daylight and in darkness. Defensive stridulation is known from many species of arachnids, myriapods, insects, as well as crustaceans. Usually, these species use stridulation to warn predators against inflicting an attack on poisonous scorpions, spiders, harvestmen, centipedes, or mutilid wasps (Iorio 2003; Pomini et al. 2010; Esposito et al. 2018; Gall et al. 2018; Stidham 2019). The sounds could therefore fulfil the same role as gaudy colouration (Rowe 2002). Stridulation also can be mimicked e.g., harmless spiders which mimic mutilid wasps in size, colour, and stridulation (Pekár et al. 2020), though this is rarely used as part of the behaviour of a mimicry model. Although we do not have any evidence about a possible mimicry model for *A.officinalis*, which can teach predators to avoid dangerous vibrating prey, stridulation can be effective as a defensive behaviour without a painful experience for the predator. Vibrating can cause prey to be dropped, resulting in its loss on the soil surface. The ball-like shape of conglobated stridulating *Armadillo* makes it predestined to roll away. However, experiments with experienced and naïve predators of *A.officinalis* should be done to confirm this theory.

## Not to be edible

When a terrestrial isopod is found and recognised by a predator, there are some other possible strategies it can use to avoid being consumed. A widespread strategy to repulse predators is the use of excretions from the defensive glands. A detailed description of the glands of terrestrial isopods, which are diverse and numerous, was done by Gorvett (1946, 1951, 1952, 1956). There are several different kinds of glands, occurring in almost every part of the body. As pointed out by Gorvett (1951), lobed glands are the largest, as well as the most interesting, of the tegumental glands. They have numerous long ducts that end in separate external openings along the lateral plates and uropods. After strong stimulation, visible droplets of a viscous, smelly secretions appear from these openings. The distribution and size of these glands were studied by Herold (1913), who explained the reduction of their function in some myrmecophilous species, that are defended by tenant ants. On the other hand, the reduction of the function of these glands can be a reason for ant adoption of some myrmecophilous species, as they are chemically insignificant for them (Parmentier 2016), and difficult to recognise in an ant nest.

**Figure 1.**
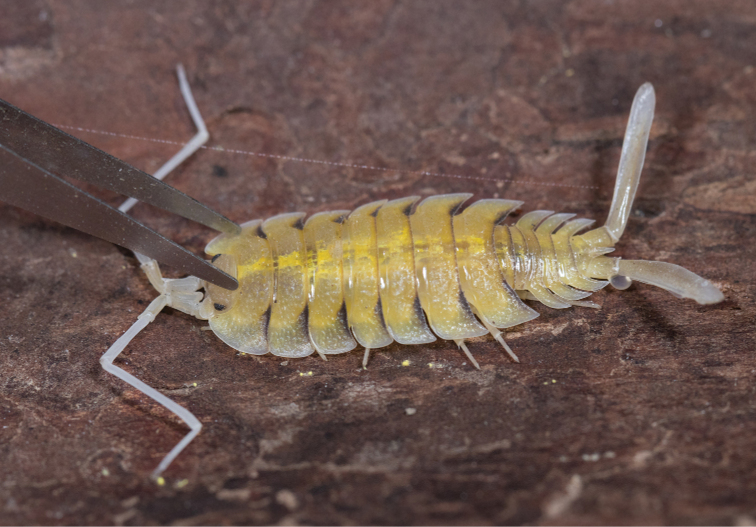
A male of *Porcelliobolivari* Dollfus, 1892 releasing proteinaceous secretion from the uropods (string with droplets) (photograph Adrián Purkart).

The substance of lobed glands has a proteinaceous composition with a secretion that is not associated with the hormonal or nervous system; it is instead probably caused by the contraction of adjacent muscles. The stimulation must be very violent: simple shaking or squeezing of the animal does not affect gland secretion, in general (Fig. 1). Gorvett (1956) experimentally confirmed that droplets of secretion appeared after a spider bit a woodlouse on the leg, or after an experimental pinprick. It is apparent, then, that the function of these glands is to produce defensive secretions against spiders that belong to the most significant predators of terrestrial isopods. The pores of the lobed glands are in an optimal position for maximal effect against attacking spiders (Gorvett 1956), centipedes (Paris 1963), or ants (Deslippe et al. 1996). After a spider bites, using its chelicerae, lobed glands begin to secrete defensive secretions, causing the predator to retreat in order to clean its mouth parts. This observation is supported by the fact that terrestrial isopods with missing parts of the uropods or lateral parts are frequently found in nature. This may be caused by shrews’ (Brereton 1957) or scorpions’ (Herold 1913) incomplete attacks (Gorvett 1956).

Instead of discouraging a predator from an attack using repellent glands, terrestrial isopods may display unpalatability from a distance in order to avoid risking damage. One way to do this is by using a warning aposematic colouration. This strategy is widely adopted by different insects, millipedes (Marek and Bond 2009), and arachnids. Warning colours (usually black combined with red, orange, or yellow) can warn predators of their unpalatability through inherited neophobia or learned avoidance for these colours (Vickers et al. 2021). So far, we have only anecdotal evidence for the aposematic function of colouration in terrestrial isopods. Levi (1965) compared bright red spots on *Armadillidiumklugii* Brandt, 1833, the millipede *Glomerispulchra* CL Koch, 1847, and the widow spider *Latrodectustredecimguttatus* (Rossi, 1790), all living syntopically near Dubrovnik. All of these species defend themselves chemically, that support their Mullerian mimicry system against attacks from thrushes, gallinaceous birds, nocturnal mammals, and geckos (Schmalfuss 2013).

**Figure 2.**
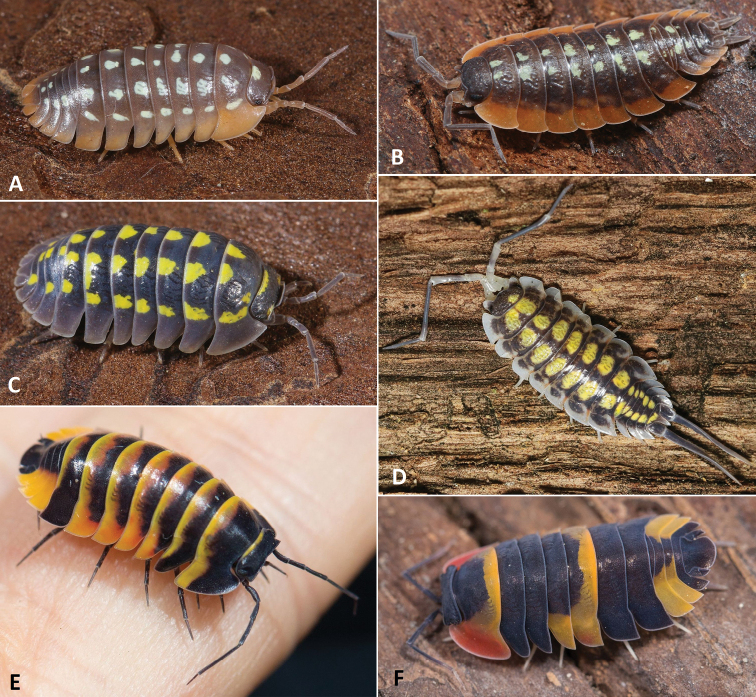
Colourful species of terrestrial isopods: **A***Armadillidiumwerneri* Strouhal, 1927 **B***Porcellioduboscqui* Paulian de Félice, 1941 **C***Armadillidiumgestroi* Tua, 1900 **D***Porcelliohaasi* Arcangeli, 1925 **E** “*Merulanella*” sp. 1 from Thailand **F** “*Merulanella*” sp. 2 from Thailand (photographs Adrián Purkart).

There are dozens of species with an ostentatious colouration (Fig. 2). Yellow and white spots are quite common in the genus *Porcellio*, but “black” and white patterns (more precisely dark violet-brown and white patterns) were reported for several non-related litter-dwelling species in western Africa (Schmalfuss and Ferrara 1982). Its antipredation function was suggested to not only be a warning colouration, but also a cryptic colouration that dissolves the body outline (Schmalfuss and Ferrara 1982). Some species are capable of polychromatism (Achouri and Charfi-Cheikhrouha 2009), in which individuals with different colour patterns coexist in the same population. Such variability in colour pattern can be useful for individuals having less frequent variations since predators are generally attracted to the most frequent prey type due to perceptual learning (“search image”, see Punzalan et al. 2005). Thus, polychromatism is a mechanism of negative frequency-dependent selection, where a rare morph prey experiences a higher survival rate than those of more common types (Hughes and Mather 1986).

Warning colouration works only against visual predators, but there could be another possible method to warn other predators: spines on the dorsal surface of terrestrial isopods. Extravagant pin-like or blade-like spinulation is typical for several species of the families Armadillidae (species of the genera *Pseudolaureola*, *Calmanesia*, *Echinodillo*, *Tridentodillo*, *Globarmadillo*, *Polyacanthus*), Eubelidae (*Panningillo*), and Delatorreidae (*Pseudarmadillo*, *Acanthoniscus*). All of these species are able to conglobate, and are of small size (~ 1 cm at maximum).

**Figure 3.**
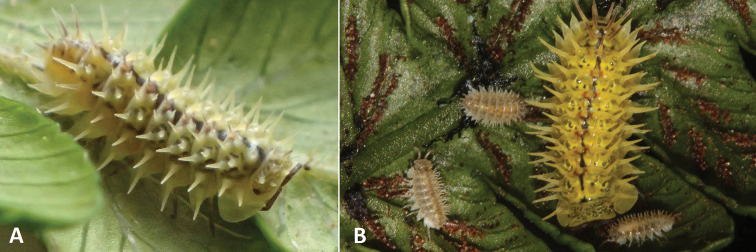
The spiky yellow woodlouse *Pseudolaureolaatlantica* (Vandel, 1977), endemic to St. Helena Island **A** its bright coloration and spines can serve as protection from potential predators (photograph Amy-Jayne Dutton, St Helena National Trust) **B** juveniles of *P.atlantica* are well protected as soon as they leave the marsupium, and remain close to their mother (photograph Phil Lambdon).

Long spines on terrestrial isopods can also, theoretically, be useful against predators (Fig. 3). The spiky yellow isopod, *Pseudolaureolaatlantica* (Vandel, 1977), lives on tree fern leaves, and, despite its vivid colour, softens its body outline with its long spines (Dutton and Pryce 2018). The function of long spines has not been studied to date, but it is assumed that spines protect terrestrial isopods against swallowing by geckos and other lizards, frogs, and birds, as well as from ants and other smaller predators (Schmalfuss 1975). Among millipedes, soft-bodied bristly millipedes (Polyxenida) without defence glands wear lateral tufts of setae and use them against ants (Eisner et al. 1996). Perhaps long spines on some small conglobating terrestrial isopods can prevent them against grasping by ant mandibles. A similar function can be ascribed to shorter and stronger thorns, which are more frequent among terrestrial isopods. Strong thorns can also protect larger soil-dwelling terrestrial isopods. The genus *Hemilepistus* (e.g., *Hemilepistusaphganicus* Borutzky, 1958) has strong thorns on the anterior part of the head and posterior margin of the first four pereonites. The function of this armature is to plug the entrance of its burrow, and to protect the individuals inside against small predators and other intruders (Schmalfuss 1975).

**Figure 4.**
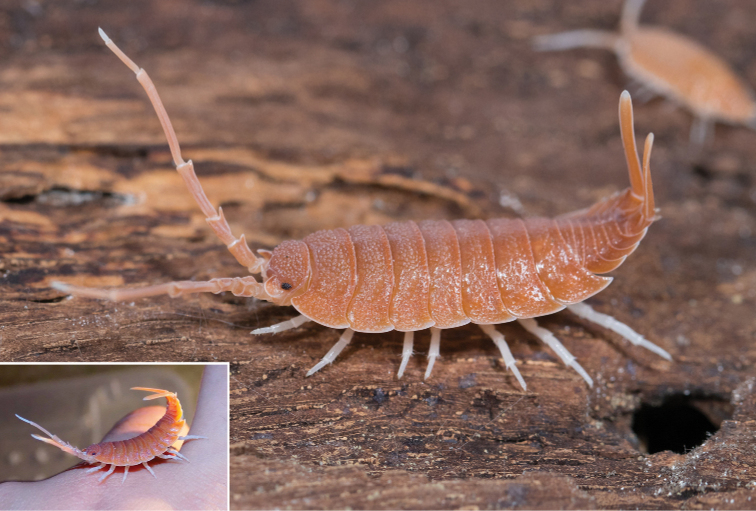
Threatening posture of a male of *Porcelliomagnificus* Dollfus, 1892 resembling the threatening posture of a syntopic *Buthus* scorpion (photographs Adrián Purkart).

A threatening posture, as seen in scorpions, bird-spiders, or centipedes, is another warning signal that protects prey from predators (Kronmüller and Lewis 2015). Several large species of *Porcellio* from the Iberian peninsula are capable of bending the posterior part of their body upward, with their long uropods targeted forward, and with widely outspread antennae (Fig. 4) when they are disturbed. This posture resembles the posture of a scorpion. The Iberian peninsula is inhabited by at least a dozen species of the scorpion genus *Buthus* (Teruel and Turiel 2020), all of which have pale orange-brown colour and thin chelae. *Porcelliomagnificus* Dollfus, 1892 is of orange colour and readily takes this posture. Although we lack supporting experimental evidence about whether this behaviour can avert predator attacks, it is known that even some lizards from Southern America (Brandão and Motta 2005) and Asia (Autumn and Han 1989) are known to be scorpion mimics. A scorpion-like threatening posture is not possible to use in a tight shelter or a burrow but only at the surface. Due to probable nocturnal activity, as well as its only superficial resemblance, it is plausible to suppose that *Porcellio* can use this posture during moonlit nights against their predators. However, this topic has not been studied yet.

## Conclusions

We have summarised what is known about the antipredatory strategies of terrestrial isopods. Some anatomical and behavioural traits should be classified as pre-adaptations because they help to solve other challenges of woodlouse life, such as the reduction of water loss. Examples include conglobation and aggregation, as well as clinging (Warburg 1993). Additionally, some proposed strategies can execute different functions, e.g., long spines can be used as tactile sensors or for collecting water from fog as well as defence mechanism. On the other hand, the significance of the jumping or scorpion-like threatening posture is difficult to understand without considering the behaviour of its predators. Some of the strategies mentioned above, such as escape by turn alternation, tonic immobility, production of repelling secretions, or conglobation were studied with their respect to predation pressure, but most of them were only suggested to have this protective function against predators. These knowledge gaps deserve attention in future research.
